# Effects of Irritant Chemicals on *Aedes aegypti* Resting Behavior: Is There a Simple Shift to Untreated “Safe Sites”?

**DOI:** 10.1371/journal.pntd.0001243

**Published:** 2011-07-26

**Authors:** Hortance Manda, Luana M. Arce, Tarra Foggie, Pankhil Shah, John P. Grieco, Nicole L. Achee

**Affiliations:** Department of Preventive Medicine and Biometrics, Uniformed Services University of the Health Sciences, Bethesda, Maryland, United States of America; Centers for Disease Control and Prevention, United States of America

## Abstract

**Background:**

Previous studies have identified the behavioral responses of *Aedes aegypti* to irritant and repellent chemicals that can be exploited to reduce man-vector contact. Maximum efficacy of interventions based on irritant chemical actions will, however, require full knowledge of variables that influence vector resting behavior and how untreated “safe sites” contribute to overall impact.

**Methods:**

Using a laboratory box assay, resting patterns of two population strains of female *Ae. aegypti* (THAI and PERU) were evaluated against two material types (cotton and polyester) at various dark:light surface area coverage (SAC) ratio and contrast configuration (horizontal and vertical) under chemical-free and treated conditions. Chemicals evaluated were alphacypermethrin and DDT at varying concentrations.

**Results:**

Under chemical-free conditions, dark material had significantly higher resting counts compared to light material at all SAC, and significantly increased when material was in horizontal configuration. Cotton elicited stronger response than polyester. Within the treatment assays, significantly higher resting counts were observed on chemical-treated dark material compared to untreated light fabric. However, compared to matched controls, significantly less resting observations were made on chemical-treated dark material overall. Most importantly, resting observations on untreated light material (or “safe sites”) in the treatment assay did not significantly increase for many of the tests, even at 25% SAC. Knockdown rates were ≤5% for all assays. Significantly more observations of flying mosquitoes were made in test assays under chemical-treatment conditions as compared to controls.

**Conclusions/Significance:**

When preferred *Ae. aegypti* resting sites are treated with chemicals, even at reduced treatment coverage area, mosquitoes do not simply move to safe sites (untreated areas) following contact with the treated material. Instead, they become agitated, using increased flight as a proxy indicator. It is this contact irritant response that may elicit escape behavior from a treated space and is a focus of exploitation for reducing man-vector contact inside homes.

## Author Summary

*Aedes aegypti*, the primary vector mosquito of dengue virus, typically lives near or inside human dwellings, and feeds preferentially on humans. The control of this mosquito vector remains the most important dengue prevention method. The use of chemicals at levels toxic to mosquitoes is currently the only confirmed effective adult vector control strategy with interventions usually applied following epidemic onset. However, research indicates that sub-lethal chemical approaches to prevent human-vector contact at the house level exist: contact irritancy and spatial repellency. The optimum efficacy of an intervention based on contact irritant actions of chemicals will, however, require full knowledge of variables that will influence vector resting behavior and thereby chemical uptake from treated sources. Here we characterize the resting patterns of female *Ae. aegypti* on two material types at various dark:light surface area coverage ratios and contrast configurations under chemical-free and treated conditions using a laboratory behavioral assay. Change in resting behavior between baseline and treatment conditions was quantified to determine potential negative effects of untreated surfaces (“safe sites”) when irritant responses are elicited. We show that treatment of preferred resting sites with known irritant compounds do not stimulate mosquitoes to move to safe sites after making contact with treated surfaces.

## Introduction

Dengue, primarily transmitted by *Aedes aegypti* (L.) (Diptera: Culicidae), is presently the most important mosquito-borne viral disease in the world with over 100 countries endemic, mostly in the tropics and subtropics [1], and an estimated 2.5 billion people at risk of infection. There is no vaccine against dengue and there are no drugs to treat dengue hemorrhagic fever and dengue shock syndrome. Hence, vector control remains the cornerstone for the prevention and control of dengue transmission [2].

Patterns of dengue virus transmission are influenced by the abundance, survival, and behavior of the principal mosquito vector, *Ae. aegypti*. Two main emphases for *Ae. aegypti* control exist: (1) reduction of the larval stage through environmental management (source reduction), larvicides and biological control; and (2) reduction of the adult stage using fumigation and/or residual spray of insecticides. Since the early 1900s [3], [4], it has been known that the most cost-effective means of preventing mosquito-borne disease is to target the adult vector, which transmit the pathogen. However, the prevailing paradigm for suppressing *Ae. aegypti* targets immature mosquitoes, the vast majority of which will not survive long enough to transmit virus [5]. For emergency interventions during dengue outbreaks, targeting the adult vector population by outdoor ultra-low-volume (ULV) application of insecticides and/or indoor thermal fogging remain the methods of choice [6], [7]. However, most control interventions that apply adulticides by space-spraying achieve relatively low effectiveness [8]–[13]. One reason for this reduced effectiveness can be attributed to vector behavior. *Aedes aegypti* is extensively adapted to exploit the human environment. The female almost exclusively takes blood from humans [14] and most commonly feeds and rests indoors. This species will also lay eggs in available oviposition and larval developmental sites inside the home [15]. This extensive use of the human indoor environment poses unique challenges to traditional adult control methods since chemical applied through outdoor and peridomestic ULV methods must pass through house portals to reach the interior space where the vector can make contact with the insecticide. This approach results in the loss of some chemical prior to reaching the interior space. Control in buildings usually accomplished with indoor residual or space spray are often hampered by limited access into homes and resource limitations [5]. On the other hand, *Ae. aegypti*'s high affinity for the human indoor environment also provides opportunities for innovative approaches to control the adult vector [5].

*Aedes aegypti* has been characterized as having specific resting preferences based on visual cues (i.e., dark colors) [16], [17], and to be significantly attracted by black [18], yellow, orange and red colors [19]. Studies that have exploited *Ae. aegypti*'s attraction to color contrast (i.e. simultaneous presentation of two colors, one which mosquitoes are attracted to in order to direct them to a target) have led to the development of host-seeking adult traps such as the Fay-Prince [20], counterflow geometry trap [21], and the BG Sentinel™ trap [22]. Previous studies in Thailand [23] demonstrated the utility of exploiting the resting preference of *Ae. aegypti* to develop attractant resting boxes for quickly sampling the indoor-resting population of this species. However, in relation to world-wide dengue burden, relatively few laboratory-controlled studies have been performed to quantify these behavioral patterns, and minimal research has been conducted to determine how to exploit this knowledge to reduce *Ae. aegypti* mosquito densities inside homes where man-vector contact is high [23].

A full description of mosquito behavior provides important information on their role as disease vectors and could serve as the basis for their control. There is growing consensus that the scarce resources available for mitigating tropical public health problems should be utilized in an evidence-based and cost-effective manner [24]. Historically, adult mosquito control using fumigation and indoor residual spray has focused mainly on the lethal actions of chemicals [6], [7]. However, research shows that there are other chemical actions that break vector-human host contact [25]–[32]. Two such actions are initiating a spatial repellent or deterrent effect, thereby preventing mosquito entry into a treated space (house); and a contact irritant effect, causing an escape response from a treated space prior to mosquitoes biting humans [32]–[35].

Such non-lethal chemical approaches are being evaluated in the development of a Push-Pull strategy for *Ae. aegypti* control currently in the proof-of-concept stage in both Peru and Thailand. “Push-Pull” is defined here as a strategy that aims to (1) prevent mosquito entry into homes through repellency and/or promote their early exit from homes through contact irritancy (Push); and (2) trap repelled and/or irritated mosquitoes in the outdoor environment using peridomestic traps (Pull). The goal of the strategy is to target preferred mosquito house entry portals and/or indoor resting sites with standard vector control chemicals (i.e. chemicals approved by World Health Organization Pesticide Evaluation Scheme for use in vector control) to make them unsuitable. The approach is to use minimum effective chemical dose and treated surface area coverage to reduce indoor densities of host-seeking (i.e., female adult) *Ae. aegypti* populations. One component to achieving this goal includes quantifying the patterns of resting behavior of *Ae. aegypti* exposed to chemical-free and chemical-treated surfaces to define the impact of untreated surfaces on irritancy behavior -is there a shift to resting on “safe-sites” resulting in an attenuated escape response?

The overall aim of the current study was to use a simple laboratory assay to characterize the resting patterns of two geographically distinct female *Ae. aegypti* population strains in response to material texture (cotton and polyester), at varying dark:light color surface area coverage ratios, using different fabric contrast configuration (horizontal and vertical) under chemical-free (baseline), and chemical-treated conditions against alphacypermethrin and DDT. Change in resting behavior between baseline and treatment conditions was quantified in order to determine the potential impact of safe-sites to the contact irritant response.

## Methods

Mosquito rearing procedures, laboratory assay device structure and resting behavior test protocols can be found at www.usuhs.mil/pmb/gsvc.

### Mosquitoes

Two *Ae. aegypti* test populations (F_2_–F_5_ generations) were used: one from Pu Teuy Village, Kanchanaburi Province, Thailand (THAI) and the other from Iquitos, Peru (PERU). Larvae were reared from eggs shipped to the Uniformed Services University of the Health Sciences (USUHS), Bethesda, USA from Kasetsart University, Bangkok, Thailand or the Naval Medical Research Centre Detachment (NMRCD) Iquitos Entomology Laboratory, Iquitos, Peru. At USUHS, all eggs were vacuum-hatched and larvae were sorted into groups of 50 then maintained at 28°C and 80% RH on a 12D∶12L cycle following previously established protocols [36]–[38]. Female pupae were manually sorted from male pupae based on size, and groups of 250 were placed into 1-gallon plastic containers and allowed to emerge to adults. Females (5–7 days old) were maintained with sugar pads saturated in a 10% sucrose solution until 24-hour prior to day of testing. The USUHS colonies were maintained until the F_5_ generations then refreshed with corresponding F_1_ field material to help ensure comparability between laboratory and field populations.

### Laboratory assay device

**Figure 1:**
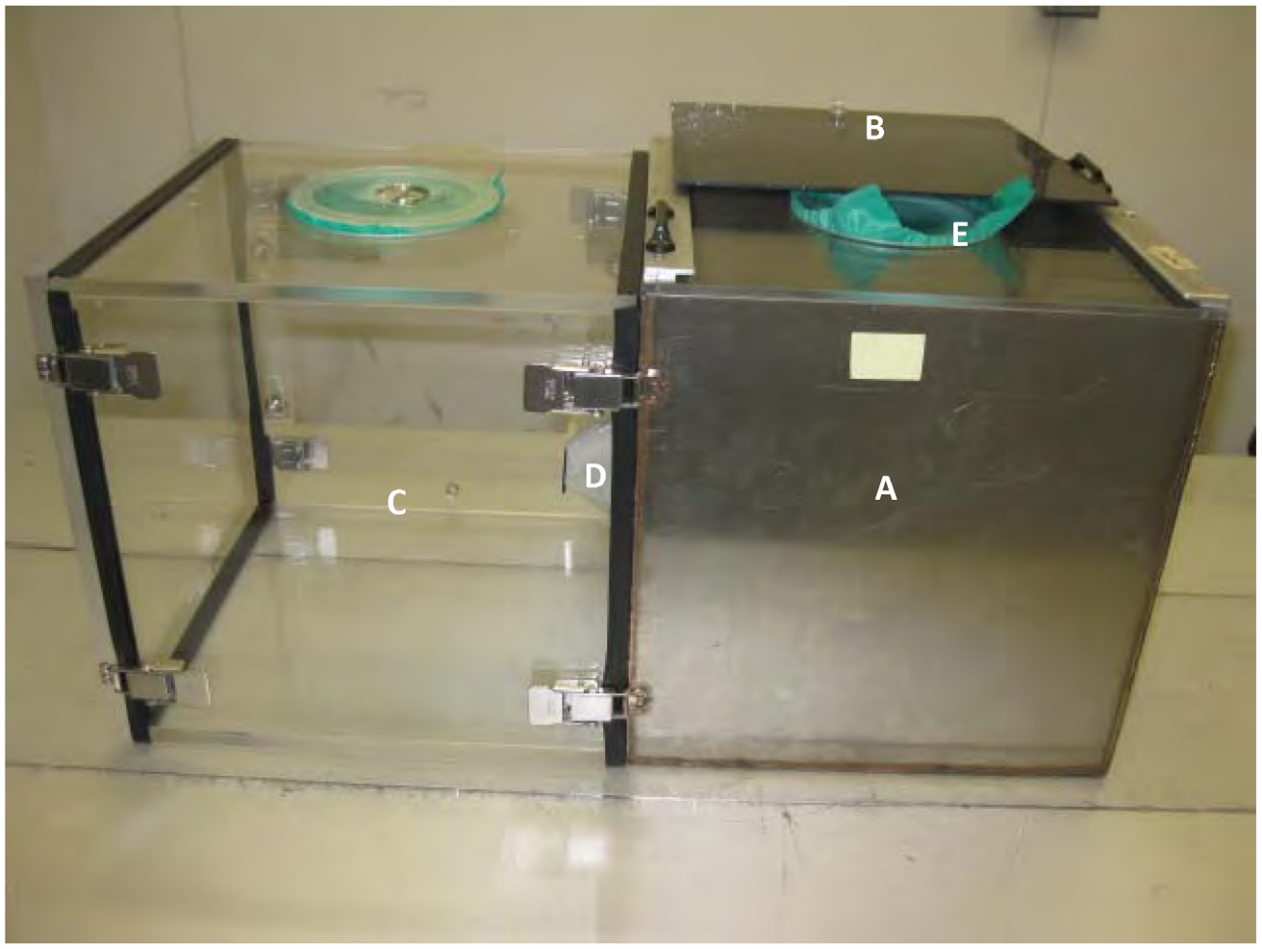
Laboratory assay device used to evaluate *Ae. aegypti* resting behavior. (A) treatment Box, (B) viewing lid, (C) Plexiglas chamber for movement evaluation, (D) funnel gate, (E) mosquito introduction/removal portal.

The laboratory assay device (i.e.“Box Assay”) is a modular system based on the HITSS [36] and excito-repellency test chamber [39]. It is composed of metal and Plexiglas boxes (30×30 cm) that can be joined together using metal hinges (Figure 1). The main test chamber contains material pieces (either chemical-free or treated) while the Plexiglas box can be added to quantify spatial repellency (i.e., reduced entry) or contact irritancy (i.e., increased exiting) during mosquito movement studies. For the purpose of this study, only the metal test boxes were used. The metal test box is fitted with a Plexiglas lid to facilitate observation of mosquito behavior during testing. The Plexiglas lid contains a portal covered with dental dam through which mosquitoes are introduced at the beginning of a test replicate and removed following the last observation. The Plexiglas lid can be covered with a sliding tinted cover that can be opened during observational time points and closed afterwards to maintain darkness in the box throughout the rest of the test procedure.

### Chemicals and treatment procedures

Chemicals evaluated in this study, DDT and alphacypermethrin, were chosen based on current status of World Health Organization Pesticide Evaluation Scheme (WHOPES) residual chemical recommendations and/or historical use in vector control programs [40]. Chemicals were acquired as technical grade material purchased from Sigma-Aldrich (St. Louis, MO): DDT (CAS 50-29-3), alphacypermethrin (CAS 67375-30-8). For resting experiments with chemical, dark material strips were treated with various doses (2.5; 25 and 250 nmol/cm^2^) of alphacypermethrin or DDT diluted in acetone solution. Assay concentrations were selected according to previous behavioral studies with these chemicals [32], [33], [36]. Treatment solutions were applied evenly to individual material strips using a micropipette. Additional material strips were treated with acetone solvent to serve as untreated controls. All fabric pieces were treated approximately 30 min prior to initiating the first replicate of the assays and allowed to air-dry on a drying rack for at least 15 min before being inserted into the metal test boxes. New treatment and control material strips were prepared daily.

### Observations of resting patterns

**Figure 2:**
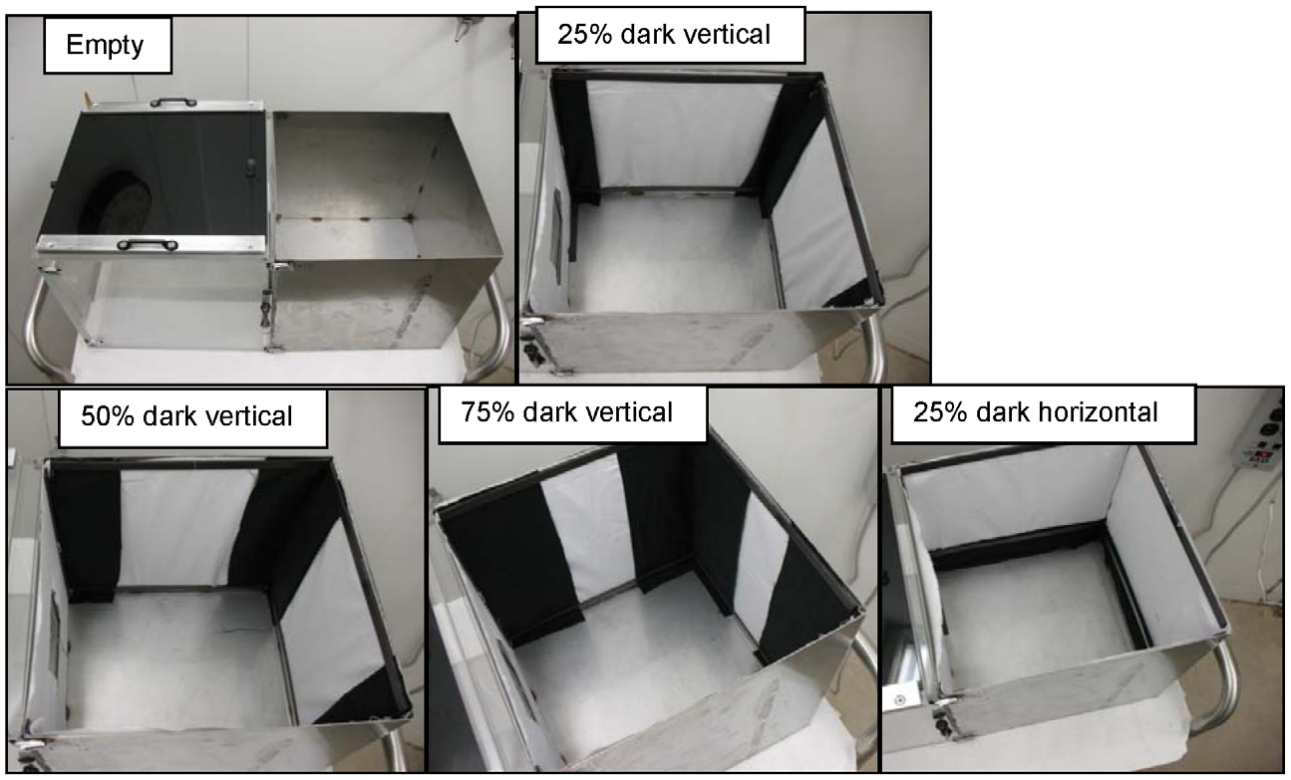
Varying surface area coverage ratios of dark:light material in both vertical and horizontal configurations.

The materials used in the resting behavior studies consisted of either black or white cotton (Natural Charm 43/44” wide 100% cotton 68×68 D/R-black and white, Bruce Variety, Bethesda, MD, USA); and green or white 100% polyester netting (BioQuip Products, Rancho Dominguez, CA, USA; mesh size 24×20/inch). Three variables that could influence resting behavior were evaluated for each *Ae. aegypti* strain: (1) surface area coverage (SAC) ratio of dark to light material; (2) vertical versus horizontal configuration of dark fabric strips; and (3) material texture. A total of six replicates were performed at each dark : light coverage ratio and contrast configuration (horizontal and vertical) for both material types (cotton and polyester) under chemical-free and treated conditions. Preference for upper versus lower positioning was also recorded during horizontal configuration studies. A chalk line was used to discern “upper” versus “lower” regions during 100% coverage experiments. Depending on the experiment type, chemical-free (control and baseline assays) and chemical-treated (treatment assays) cotton or polyester panels were placed into corresponding metal test boxes at 100% dark, 100% white, 75%∶25%, 50%∶50%, and 25%∶75% dark : light (D:L) SAC ratios (Figure 2). All material panels were attached to the assay walls using magnets. For each test assay, a matched control with chemical-free (solvent-treated) dark material was performed simultaneously.

Separate groups of 10 females were introduced into the metal test box and counts made of resting locations every 2 minutes, during a 10 minute sampling period. Six replicates were performed for each test type. The four sides of the metal box were designated as rear, front, right and left, facilitating the recording process. In each test, resting locations were recorded as: 1) dark or light material; 2) magnet; 3) floor and; 4) Plexiglas lid. In addition, the number of mosquitoes flying inside the metal box, and those knocked down (KD) (defined as lying on their side or back and unable to right themselves when the test box was gently tapped) were also tallied. All testing was performed under controlled temperature (28–30°C) and relative humidity (50–60%). For assays containing chemical treatments, test boxes were cleaned at the end of each day of testing with acetone and allowed to air-dry overnight before reuse with a new chemical or a different chemical concentration.

### Data analysis

All analyses were performed using SPSS 16.0 and SAS 9.2.

Counts made at each 2 min observation period from all six replicates for each test assay were summed and calculated as the proportion of mosquitoes observed resting for each of the specified observation locations as well as those exhibiting flying and knockdown responses. Comparisons among observations within a single test box (control and treatment) were performed using chi-square statistical analyses (observed versus expected resting on dark or light material) with a 2×2 contingency table at a 95% confidence level.

The effects of material configuration were determined by comparing the difference in the proportion of mosquitoes resting on the dark material strips placed horizontally versus those resting on the strips placed vertically. For horizontal configuration experiments, proportions resting on upper and lower dark strips were also compared. The effect of texture was determined by comparing proportions resting on dark cotton versus dark polyester in a separate test. Percent change in resting on treated dark surfaces and untreated surfaces (using proportions resting on untreated light material, KD and flying as indicators) were quantified by comparing proportions observed in the treatment box with matched controls (chemical-free condition). Pearson correlations were also used to determine relationships between chemical test dose and change in resting behavior and/or proportion flying; and relationships between SAC and change in resting behavior and/or proportion flying at each chemical test dose.

## Results

### Resting patterns under chemical-free conditions

#### Dark versus light material

For experimental tests using 100% dark or 100% light material, results indicated that overall resting on material versus other surfaces within the test box was improved when 100% dark (black cotton or green polyester) was used versus 100% light material (white). This was significant for both THAI (95.5% vs. 84.4% and 90.0% vs. 84.7% dark vs. light with cotton and polyester, respectively) and PERU (93.3% vs. 82.7% and 95.2% vs. 87.9% dark vs. light with cotton and polyester, respectively) strains (*P<*0.05). Results for both *Ae. aegypti* strains show significantly higher resting observations on dark cotton and polyester strips over light (*P<*0.05) at each D:L SAC ratio and contrast configuration design (horizontal and vertical) (Table 1).

**Table 1:** Resting observations of *Ae. aegypti* on chemical-free material at varying dark:light surface area coverage.

MosquitoStrain	Material	Configu-ration	SAC(%)	Proportion observed resting (%)		P*D vs. L	P**H vs. V	P*** Cotton vs. Polyester
				Dark	Light			
THAI	Cotton	N/A	100	95.5	N/A	N/A	N/A	-
				N/A	84.3	N/A	N/A	-
		H	75	96.9	3.3	S	S	S
		V		86.3	6.1	S		S
		H	50	90.7	6.7	S	S	S
		V		73.5	18.5	S		S
		H	25	70.5	22.0	S	S	S
		V		41.2	28.6	S		S
	Polyester	N/A	100	90.0	N/A	N/A	N/A	
				N/A	85.8	N/A	N/A	
		H	75	86.1	10.9	S	S	
		V		59.4	21.8	S		
		H	50	77.0	10.9	S	S	
		V		64.9	40.4	S		
		H	25	56.1	26.6	S	S	
		V		36.0	50.7	S		
PERU	Cotton	N/A	100	93.3	N/A	N/A	N/A	-
				N/A	82.6	N/A	N/A	-
		H	75	82.7	0.0		S	S
		V		76.7	12.1	S		S
		H	50	81.9	3.8	S	S	S
		V		56.5	7.8	S		S
		H	25	79.5	12.2	S	S	S
		V		65.7	13.3	S		S
	Polyester	N/A	100	94.9	N/A	N/A	N/A	
				N/A	87.9	N/A	N/A	
		H	75	63.1	13.7	S	S	
		V		58.9	28.6	S		
		H	50	57.4	8.9	S	S	
		V		49.2	42.6	S		
		H	25	43.2	37.8	S	S	
		V		36.0	46.0	S		

*Chi-square comparison for resting on the dark versus light material at each D:L SAC.

**Chi-square comparison for resting on the dark versus light when dark material strips are on vertical versus horizontal configuration at each D:L SAC.

***Chi-square comparison for resting on the dark versus light when cotton was used versus polyester at each D:L SAC.

S  =  P<0.05; NS  =  P>0.05; N/A  =  Not applicable; -  =  Not performed; D  =  dark; L  =  light; SAC  =  surface area coverage; H  =  horizontal; V  =  vertical; N  =  60 from a total of 6 replicates performed for each assay type.

#### Effects of configuration (vertical versus horizontal) on resting patterns

For both THAI and PERU *Ae. aegypti* strains, resting observations on dark material (both cotton and polyester) was significantly increased when placed in a horizontal versus vertical configuration design. This was true at all three D:L coverage ratios (*P<*0.05) (Table 1).

#### Resting patterns on upper versus lower regions of chemical-free strips

During trials with 100% dark and 100% light surface area coverage, the tendency of THAI to rest on the upper half of walls was significant (*P<*0.05) for both colors and material types (Table S1). With PERU, similar patterns were obtained, with significantly higher observations of resting above the midway designation of the wall's height using 100% light cotton and polyester (*P<*0.05) (Table S1). Trials for evaluating horizontal configuration showed that with both strains of *Ae. aegypti* there were significantly higher resting observations made on the lower dark strips as compared to the upper strips at all three D:L SAC using cotton (*P<*0.05) (Table S1). However, the opposite was observed with polyester where significantly higher resting observations were made (*P<*0.05) on the upper dark strips as compared to the lower strips at all three D:L SAC (Table S1).

#### Cotton versus polyester material / Effects of texture on resting patterns

For both THAI and PERU, at all D:L SAC ratios, regardless of material configurations, resting observations on dark versus light material was significantly increased when cotton was used versus polyester in the assays (*P<*0.05) (Table 1). In order to determine if material texture might be confounding the behavioral response, resting patterns were observed using the THAI strain against all SAC ratios using green : white cotton and green : white polyester simultaneously in two separate text boxes. Results indicated higher proportions of mosquitoes resting on dark cotton compared with dark polyester. This was significant at 25% dark coverage in vertical (Table S2), and both 25 and 75% dark coverage in the horizontal configuration (Table S2) (*P<*0.05).

### Resting patterns under chemical-treatment conditions

#### Dark treated material versus untreated light material

When dark material was treated with chemical, results indicated significantly more resting than expected on the dark treated material compared to the untreated light material within the assay chamber. This was true for cotton material at all D:L coverage for both chemicals at all test doses (Tables S3, S4, S5). However, significantly fewer mosquitoes were observed resting on the insecticide-treated dark material overall compared to chemical-free dark material in the matched control assays. This pattern was true for both *Ae. aegypti* strains and for all doses of both chemicals applied to either cotton or polyester material (Tables 2, 3, 4 and 5). More importantly, resting observations on safe-sites (untreated light material) in the treatment test chamber did not significantly increase for the majority of tests to include the 25% SAC (Tables 2, 3, 4, and 5). Knockdown rates were ≤5% for all assays (Tables 2, 3, 4, and 5), with significantly more observations of flying mosquitoes made in the test box under chemical-treatment conditions as compared to the chemical-free matched control (Tables 2, 3, 4, and 5).

**Table 2:** Resting observations of *Ae. aegypti* THAI strain within both treatment (alphacypermethrin) and matched control conditions.

alphacypermet hrin doses(nmol/cm^2^)	Material	Configu-ration	SAC(%)	*Ae. aegypti* THAI strain: Proportions observed resting on dark, light/KD/Flying (%)										
				Dark			Light			KD		Flying		
				1 ^,^ 2 ^,^ 3Treat.	Cont.	*P	1 ^,^ 2 ^,^ 3Treat.	Cont.	*P	1 ^,^ 2 ^,^ 3Treat.	Cont.	1 ^,^ 2 ^,^ 3Treat.	Cont.	*P
2.5	Polyester	N/A	100	63.3	92.8	S	N/A	N/A	N/A	0.0	0.0	31.0	0.4	S
				N/A	N/A	N/A	47.6	91.5	S	0.0	0.0	45.9*^a^*	0.02	S
		H	75	50.8*^a^*	84.3	S	0.07^b^	0.05	NS	0.03	0.01	26.9^a^	0.01	S
			50	34.1*^a^*	81.0	S	26.5^b^	13.8	NS	2.3	0.0	26.8^a^	0.0	S
			25	34.6^a^	62.5	S	39.7^a^	24.6	S	0.0	0.0	18.3^a^	0.0	S
		V	75	38.0^b^	57.6	S	17.3*^a^*	31.9	S	0.01	0.0	36.7^b^	0.03	S
			50	26.8^b^	64.4	S	25.2^a^	23.7	S	0.04	0.0	33.8^a^	0.0	S
			25	28.7^a^	47.7	S	49.8*^b^*	43.2	NS	0.0	0.0	16.3^a^	0.02	S
25	Cotton	N/A	100	79.7	96.0	S	N/A	N/A	N/A	0.0	0.0	16.0	16.7	S
				N/A	N/A	N/A	26.1	74.6	S	0.3	0.0	58.6	0.0	S
		H	75	70.7*^a,A^*	91.5	S	3.4^b,B^	1.7	NS	0.0	0.0	21.1^b, A^	0.0	S
			50	55.2*^a,^* ^A^	80.9	S	3.8^b, A^	7.1	NS	0.7	0.0	17.2*^a,^* ^B^	0.0	S
			25	50.8^a,A^	70.5	S	26.1^b, B^	22.0	NS	0.0	0.0	16.1^a, A^	0.3	S
		V	75	35.6^b,A^	71.3	S	9.7*^a,^* ^B^	10.9	NS	0.0	0.0	38.1*^a,A^*	3.4	S
			50	44.8^b,*A*^	66.1	S	19.1*^a,^* ^B^	12.6	S	0.0	0.0	14.0^b, B^	3.8	S
			25	44.7^a,*A*^	75.9	S	35.3*^a,^* ^B^	15.5	S	0.0	0.0	12.3^a, A^	1.7	S
	Polyester	N/A	100	49.3	89.8	S	N/A	N/A	N/A	0.7	0.0	37.8	0.0	S
				N/A	N/A	N/A	35.4	91.9	S	0.0	0.0	44.4	0.0	S
		H	75	37.3*^a,^* ^B^	85.2	S	9.2^b, *A*^	3.7	S	5.9	0.0	23.1^a, A^	0.7	S
			50	55.2*^a,^* ^A^	80.9	S	3.8^b, A^	7.1	NS	0.7	0.0	17.2^a, *A*^	0.0	S
			25	33.6*^a,^* ^B^	65.0	S	34.5^b, *A*^	28.3	NS	0.0	0.0	26.2^a, A^	0.0	S
		V	75	36.7^b,A^	71.0	S	36.0*^a,A^*	8.6	S	0.0	0.0	21.6^a, B^	6.9	S
			50	24.5^b,B^	59.3	S	42.0*^a, A^*	32.1	S	0.7	0.0	21.0^a, *A*^	1.7	S
			25	9.7^b,B^	28.8	S	45.0*^a, A^*	33.7	S	0.7	2.5	16.4^a, A^	6.3	S

*^χ2^test P comparing resting on the dark material under treatment versus matched control conditions. Treat.  =  Treatment; Cont.  =  Control.

1 Small capital letter compares resting observation in dark:light; KD and mosquitoes observed flying when dark material are in vertical versus horizontal configuration in treatment conditions. Same small capital letter on the same column at the same SAC, material type, chemical and dose means percent observed resting not significantly different.

2 Capital letter compare resting observation in dark:light, KD and mosquitoes observed flying when cotton is used versus polyester in treatment conditions. Same capital letter on the same column at the same SAC, configuration, chemical and dose means percent observed resting not significantly different.

3 Letter in italic refers to the value significantly high.

S  =  P<0.05; NS  =  P>0.05; N/A  =  Not applicable; SAC  =  surface area coverage; H  =  horizontal; V  =  vertical; N  = 60 from a total of 6 replicates performed for each assay type.

**Table 3:** Resting observations of *Ae. aegypti* THAI strain within both treatment (DDT) and matched control conditions.

DDT doses(nmol/cm^2^)	Material	Configu-ration	SAC(%)	*Ae. aegypti* THAI strain: Proportions observed resting on dark, light/KD/Flying (%)										
				Dark			Light			KD		Flying		
				1 ^,^ 2 ^,^ 3Treat.	Cont.	*P	1 ^,^ 2 ^,^ 3Treat.	Cont.	*P	1 ^,^ 2 ^,^ 3Treat.	Cont.	1 ^,^ 2 ^,^ 3Treat.	Cont.	*P
25	Cotton	N/A	100	70.6	93.5	S	N/A	N/A	N/A	2.0	0.0	17.7	1.0	S
				N/A	N/A	N/A	45.5	69.2	S	0.0	0.0	37.8	1.7	S
		H	75	72.8^a,*A*^	83.9	S	9.5^a,A^	8.2	NS	0.0	0.0	16.1^a,A^	0.0	S
			50	67.6^a,*A*^	85.0	S	15.1^a,B^	8.3	S	0.0	0.0	15.7^a,B^	0.0	S
			25	60.4^a,*A*^	75.0	S	21.8^a,A^	21.7	NS	0.0	0.0	17.8^a,A^	0.0	S
		V	75	73.3^a,*A*^	81.4	S	10.1^a,B^	18.6	S	0.0	0.0	14.9^a,A^	0.0	S
			50	71.5^a,*A*^	87.3	S	10.0^a,B^	8.3	NS	1.3	0.0	11.3^a,B^	0.7	S
			25	58.2^a,*A*^	83.6	S	21.2^a,B^	14.4	S	0.0	0.0	16.3^a,*A*^	0.3	S
	Polyester	N/A	100	60.1	87.5	S	N/A	N/A	N/A	0.0	0.0	28.8	0.0	S
				N/A	N/A	N/A	62.4	83.3	S	0.0	0.0	27.6	3.3	S
		H	75	42.2^a,B^	76.2	S	10.4^b,A^	3.8	S	0.0	1.7	20.9*^a,^* ^A^	5.2	S
			50	30.8*^a,^* ^B^	48.8	S	26.2^a,*A*^	11.0	S	1.7	0.0	22.4^a,*A*^	1.7	S
			25	30.0^a,B^	66.2	S	28.6^b,A^	26.6	NS	0.0	1.4	27.5*^a^* ^,A^	1.0	S
		V	75	36.6^a,B^	60.9	S	17.6*^a,A^*	16.1	NS	1.8	0.0	13.6^b,A^	3.4	S
			50	17.5^b,B^	42.7	S	32.3^a,*A*^	25.3	NS	1.0	0.0	17.2^a,*A*^	3.9	S
			25	26.0^a,B^	37.0	S	40.0*^a,A^*	38.1	NS	0.0	0.0	7.3^b,B^	2.4	S
250	Cotton	N/A	100	85.5	96.3	S	N/A	N/A	N/A	0.0	0.0	10.9	0.0	S
				N/A	N/A	N/A	67.9	81.5	S	1.8	0.0	13.1	0.3	S
		H	75	72.8^a,*A*^	83.9	S	9.5^b,*A*^	8.2	S	0.0	0.0	16.1*^a,^* ^B^	0.0	S
			50	67.6^a,A^	85.0	S	15.1^b,B^	8.3	NS	0.0	0.0	15.7*^a,^* ^A^	0.0	S
			25	60.4*^a,A^*	75.0	S	21.8^b,A^	21.7	NS	0.0	0.0	17.8^a,B^	0.0	S
		V	75	68.1^a,*A*^	80.0	S	15.9*^a,^* ^B^	8.5	S	0.0	0.0	5.1^b,B^	1.1	S
			50	58.1^a,*A*^	66.5	S	19.4*^a,^* ^B^	14.3	NS	1.7	1.7	12.8^b,A^	0.3	S
			25	42.8^b,*A*^	63.1	S	39.6*^a,A^*	29.4	S	0.0	0.0	14.7^a,*A*^	0.7	S
	Polyester	N/A	100	58.4	94.4	S	N/A	N/A	N/A	1.7	0.0	30.1	0.7	S
				N/A	N/A	N/A	63.7	74.9	S	0.0	1.0	24.4	0.3	S
		H	75	42.1*^a,^* ^B^	83.2	S	4.9^b,B^	6.1	NS	1.8	0.0	30.2*^a,A^*	1.1	S
			50	58.3*^a,^* ^A^	64.3	NS	14.2^b,*A*^	25.3	S	0.0	0.0	23.7^a,A^	0.3	S
			25	18.5^b,B^	65.9	S	21.2^a,A^	21.0	NS	0.7	1.7	33.6*^a,A^*	5.2	S
		V	75	29.1^b,B^	55.6	S	24.6*^a,A^*	10.9	S	0.0	0.0	11.2^b,*A*^	5.4	S
			50	37.8^b,B^	61.7	S	35.4*^a,A^*	28.7	NS	0.0	0.0	18.6^a,A^	2.3	S
			25	20.7*^a,^* ^B^	42.9	S	43.4^a,B^	30.4	NS	0.0	1.8	8.5^b,B^	2.9	S

*^χ2^test P comparing resting on the dark material under treatment versus matched control conditions. Treat.  =  Treatment; Cont.  =  Control.

1 Small capital letter compares resting observation in dark:light; KD and mosquitoes observed flying when dark material are in vertical versus horizontal configuration in treatment conditions. Same small capital letter on the same column at the same SAC, material type, chemical and dose means percent observed resting not significantly different.

2 Capital letter compare resting observation in dark:light, KD and mosquitoes observed flying when cotton is used versus polyester in treatment conditions. Same capital letter on the same column at the same SAC, configuration, chemical and dose means percent observed resting not significantly different.

3 Letter in italic refers to the value significantly high.

S  =  P<0.05; NS  =  P>0.05; N/A  =  Not applicable; SAC  =  surface area coverage; H  =  horizontal; V  =  vertical; N  = 60 from a total of 6 replicates performed for each assay type.

**Table 4:** Resting observations of *Ae. aegypti* PERU strain within both treatment (alphacypermethrin) and matched control conditions.

alphacypermethrin doses(nmol/cm^2^)	Material	Configu-ration	SAC(%)	*Ae. aegypti* PERU strain: Proportions observed resting on dark, light/KD/Flying										
				Dark			Light			KD		Flying		
				1 ^,^ 2 ^,^ 3Treat.	Cont.	*P	1 ^,^ 2 ^,^ 3Treat.	Cont.	*P	1 ^,^ 2 ^,^ 3Treat.	Cont.	1 ^,^ 2 ^,^ 3Treat.	Cont.	*P
25	Cotton	N/A	100	63.4	89.9	S	N/A	N/A	N/A	1.7	1.6	27.4	1.3	S
				N/A	N/A	N/A	25.1	74.6	S	3.1	0.0	57.0	4.9	S
		H	75	71.0*^a,A^*	89.8	S	0.1^b,B^	0.1	NS	0.0	0.0	22.1^a,B^	0.7	S
			50	64.4^a,*A*^	88.1	S	11.9^a,B^	10.2	NS	0.0	0.0	20.7^a,B^	0.0	S
			25	48.0^a*A*^	75.4	S	19.6^a,A^	18.0	NS	0.0	0.0	30.1^a,B^	0.0	S
		V	75	51.6^b,*A*^	85.6	S	19.0*^a,^* ^B^	11.4	S	2.6	0.0	20.3^a,B^	0.7	S
			50	59.4^a,*A*^	71.5	S	21.8^a,A^	15.3	S	0.0	0.0	14.1^a,B^	0.0	S
			25	36.6^b,*A*^	69.6	S	21.1^a,B^	9.2	S	0.0	0.0	31.5^a,*A*^	1.5	S
	Polyester	N/A	100	35.4	85.3	S	N/A	N/A	N/A	4.8	0.0	47.8	0.0	S
				N/A	N/A	N/A	32.5	81.0	S	3.0	0.0	52.6	1.3	S
		H	75	39.7*^a,^* ^B^	81.7	S	9.3^b,*A*^	7.7	NS	0.7	0.0	35.8^a,*A*^	0.0	S
			50	21.4^a,B^	73.9	S	20.1^a,*A*^	6.1	S	0.3	0.0	49.0^a,*A*^	0.0	S
			25	24.4^a,B^	45.8	S	23.1^b,A^	21.2	NS	0.0	0.0	31.4*^a,A^*	4.7	S
		V	75	26.4^b,B^	70.5	S	27.1*^a,A^*	13.1	S	0.7	0.0	31.9^a,*A*^	0.0	S
			50	20.9^a,B^	43.3	S	23.6^a,A^	40.5	S	2.3	0.0	42.5^a,*A*^	1.7	S
			25	18.7^a,B^	49.3	S	39.7*^a^* ^,*A*^	26.6	S	0.0	0.0	15.0^b,B^	1.6	S

*^χ2^test P comparing resting on the dark material under treatment versus matched control conditions. Treat.  =  Treatment; Cont.  =  Control.

1 Small capital letter compares resting observation in dark:light; KD and mosquitoes observed flying when dark material are in vertical versus horizontal configuration in treatment conditions. Same small capital letter on the same column at the same SAC, material type, chemical and dose means percent observed resting not significantly different.

2 Capital letter compare resting observation in dark:light, KD and mosquitoes observed flying when cotton is used versus polyester in treatment conditions. Same capital letter on the same column at the same SAC, configuration, chemical and dose means percent observed resting not significantly different.

3 Letter in italic refers to the value significantly high.

S  =  P<0.05; NS  =  P>0.05; N/A  =  Not applicable; SAC  =  surface area coverage; H  =  horizontal; V  =  vertical; N  = 60 from a total of 6 replicates performed for each assay type.

**Table 5:** Resting observations of *Ae. aegypti* PERU strain within both treatment (DDT) and matched control conditions.

DDT doses(nmol/cm^2^)	Material	Configu-ration	SAC(%)	*Ae. aegypti* PERU strain: Proportions observed resting on dark, light/KD/Flying (%)										
				Dark			Light			KD		Flying		
				1 ^,^ 2 ^,^ 3Treat.	Cont.	*P	1 ^,^ 2 ^,^ 3Treat.	Cont.	*P	1 ^,^ 2 ^,^ 3Treat.	Cont.	1 ^,^ 2 ^,^ 3Treat.	Cont.	*P
25	Cotton	N/A	100	79.0	94.9	S	N/A	N/A	N/A	0.0	0.0	17.0	0.0	S
				N/A	N/A	N/A	44.5	77.7	S	0.0	0.0	47.3	1.0	S
		H	75	71.2^a,*A*^	81.8	S	4.7^b,B^	13.2	S	0.0	0.0	20.0*^a,^* ^A^	0.0	S
			50	59.9^a,A^	84.3	S	11.6^a,A^	10.7	NS	0.0	0.0	26.1*^a^* ^,*A*^	0.0	S
			25	67.1^a,*A*^	78.3	S	15.9^a,A^	15.0	NS	0.0	0.0	14.0^a,B^	0.0	S
		V	75	72.8^a,*A*^	83.0	S	9.5*^a,^* ^B^	7.2	NS	0.0	0.0	12.6^b,A^	0.0	S
			50	57.9^a,*A*^	81.0	S	25.2^a,A^	13.3	S	0.0	0.0	10.3^b,B^	0.0	S
			25	58.8^a,*A*^	66.7	S	29.9^a,B^	21.3	S	0.0	0.0	10.3^a,A^	0.0	S
	Polyester	N/A	100	62.7	86.7	S	N/A	N/A	N/A	0.0	0.0	33.3	0.0	S
				N/A	N/A	N/A	65.9	82.0	S	0.0	0.0	30.7	0.7	S
		H	75	64.1^a,B^	77.7	S	17.3^b,*A*^	8.6	S	0.0	0.0	15.3*^a,^* ^A^	0.0	S
			50	69.0^a,A^	81.0	S	8.3^a,A^	11.3	S	0.0	0.0	17.0^a,B^	0.0	S
			25	30.0^a,B^	66.2	S	28.6^b,A^	26.6	NS	0.0	1.4	27.5*^a,A^*	1.0	S
		V	75	47.4^a,B^	67.7	S	17.4*^a,A^*	21.1	NS	0.0	0.0	26.0^b,A^	0.4	S
			50	35.9^b,B^	62.4	S	37.6*^a^* ^,A^	24.0	NS	0.0	0.0	16.3^a,*A*^	0.0	S
			25	26.0^a,B^	37.0	S	40.0*^a,A^*	38.1	NS	0.0	0.0	16.8^b,A^	5.7	S
250	Cotton	N/A	100	65.7	91.6	S	N/A	N/A	N/A	0.0	0.0	31.3	1.8	S
				N/A	N/A	N/A	51.7	87.1	S	0.0	0.0	35.5	0.0	S
		H	75	73.3*^a,A^*	91.7	S	5.5^b,A^	3.3	NS	0.0	0.0	16.8^a,B^	0.0	S
			50	66.6*^a^* ^,*A*^	73.8	S	13.6^a,A^	13.1	NS	0.0	0.0	15.9^b,B^	0.0	S
			25	64.3*^a,A^*	73.0	S	11.7^b,B^	18.3	S	0.0	0.0	23.0*^a,^* ^B^	0.0	S
		V	75	55.6^b,*A*^	85.4	S	16.0*^a,A^*	14.2	NS	0.0	0.0	24.3^a,A^	0.0	S
			50	46.5^b,A^	71.7	S	18.2^a,B^	23.0	NS	0.0	0.0	34.0*^a^* ^,*A*^	4.3	S
			25	47.1^b,*A*^	66.7	S	33.3*^a,^* ^B^	15.0	S	0.0	0.0	16.2^b,A^	0.0	S
	Polyester	N/A	100	58.3	85.2	S	N/A	N/A	N/A	0.0	0.0	33.7	0.0	S
				N/A	N/A	N/A	48.5	86.7	S	0.0	0.0	36.8	0.0	S
		H	75	50.0*^a,^* ^B^	79.0	S	12.7^b,A^	3.4	NS	0.0	0.0	30.7*^a,A^*	0.0	S
			50	47.5*^a,^* ^B^	70.3	S	23.3^b,A^	25.0	S	0.0	0.0	25.3*^a,A^*	0.0	S
			25	31.3*^a^* ^,B^	49.2	S	54.2^a,*A*^	40.7	NS	0.0	0.0	15.7^a,*A*^	0.0	S
		V	75	48.3^b,B^	62.7	S	13.1*^a,^* ^B^	22.0	S	0.0	0.0	23.8^b,A^	0.0	S
			50	46.3^b,A^	49.2	S	28.8*^a,A^*	42.6	NS	2.0	0.0	12.2^b,B^	0.0	S
			25	18.3^b,B^	32.8	S	54.3^a,*A*^	53.7	NS	0.0	0.0	25^a,A^	5.1	S

*^χ2^test P comparing resting on the dark material under treatment versus matched control conditions. Treat.  =  Treatment; Cont.  =  Control.

1 Small capital letter compares resting observation in dark:light; KD and mosquitoes observed flying when dark material are in vertical versus horizontal configuration in treatment conditions. Same small capital letter on the same column at the same SAC, material type, chemical and dose means percent observed resting not significantly different.

2 Capital letter compare resting observation in dark:light, KD and mosquitoes observed flying when cotton is used versus polyester in treatment conditions. Same capital letter on the same column at the same SAC, configuration, chemical and dose means percent observed resting not significantly different.

3 Letter in italic refers to the value significantly high.

S  =  P<0.05; NS  =  P>0.05; N/A  =  Not applicable; SAC  =  surface area coverage; H  =  horizontal; V  =  vertical; N  = 60 from a total of 6 replicates performed for each assay type.

There was no significant correlation (*P>*0.05) between SAC and amount of resting observed on untreated light material by mosquito strain, chemical, dose, and material type as a result of chemical exposure. Similarly, no significant correlation (*P>*0.05) was found between SAC and the proportion of mosquitoes observed flying as a result of chemical exposure for each mosquito strain using both chemicals at each dose and material type. The only exception to this being with the PERU strain against DDT 250 nm/cm^2^ using polyester (*r = *0.75, *P = *0.03); and with the THAI strain against alphacypermethrin 2.5 and 25 nm/cm^2^ using polyester (*r = *0.8, P = 0.01; and *r = *0.74, *P = *0.03 respectively).

#### Effect of vertical versus horizontal material configuration

For both THAI and PERU strains, there was a general increase in resting when chemical-treated strips were placed in the horizontal configuration (Tables 2, 3, 4, and 5). Resting on chemical-free material, however, increased when the strips were placed in a vertical configuration at most coverage ratios. In addition, at most coverage ratios and with both chemicals at all test doses, the proportion of *Ae. aegypti* observed flying was significantly higher when dark material was in a horizontal versus a vertical configuration (Tables 2, 3, 4, and 5).

#### Cotton versus polyester material/Effects of texture on resting patterns

For both strains, resting observations on the dark chemical-treated strips were significantly higher using cotton as opposed to polyester. These results were consistent for both *Ae. aegypti* strains using alphacypermethrin and DDT, at each test dose, regardless of SAC and material configuration. There were two exceptions to this which were the THAI strain when exposed to DDT 250 nmol/cm^2^ at 50% SAC in the vertical configuration, and the PERU strain when exposed to DDT 25 nmol/cm^2^ at 25% SAC in the horizontal configuration) (Tables 2, 3, 4, and 5). The proportion of mosquitoes observed resting on the untreated white material was significantly higher using polyester as compared to cotton material in most tests. Those tests that did not show this trend resulted in comparable results between materials (Tables 2, 3, 4, and 5).

## Discussion

A full understanding of adult vector ecology and behavior is vital in developing novel control strategies as well as optimizing existing tools. It is general knowledge that *Ae. aegypti* adults prefer to rest in dark, damp locations in households, and are also attracted to black colors [16]–[18] and, in fact, the development of oviposition, host-seeking and/or other adult traps are based on these observations [20]–[23], [41]–[43]. However, few standardized studies have been performed to quantify such behavioral patterns in an attempt to reduce adult mosquito densities inside homes, a site of disease transmission [23], [42].

With current suggestions that sub-lethal chemical approaches to vector control (i.e. contact irritancy) may pose viable options to reduce disease [32]–[35], [44], it is important to characterize minimal effective doses of irritant chemicals and the relationship between surface area coverage of these doses and the behavioral responses that they elicit (i.e., rapid escape from inside homes). Current adult vector control approaches such as insecticide treated bed nets, and/or clothing rely on human hosts as the attractant or bait to lure mosquitoes into contact with the treated material long enough to deliver the lethal dose of the insecticide [45]. However, when relying on the treatment of resting sites, such as the interior house walls, to reduce man-vector contact through an irritant response, interaction of the vector with these treated surfaces is facultative. Untreated areas in the house or safe-sites may be available and/or preferred for resting [12], [46] thus minimizing the impact of the intervention. It is vital therefore, to understand the drivers of these resting preferences in order to exploit and maximize the effects of irritant chemicals on vector escape responses. Such strategies will guide development of cost-effective tools for the future.

The present study quantified the resting patterns of two *Ae. aegypti* female populations (THAI and PERU) under both chemical-free and treatment conditions using a simple laboratory assay. During the chemical-free baseline trials, several variables were evaluated to include material type (cotton and polyester), dark : light color surface area coverage (SAC), and fabric configuration (horizontal, vertical). Not surprisingly, results indicate that both mosquito strains were observed resting preferentially on dark versus light colored material against both material types. These patterns were consistent using both the vertical and horizontal configuration study designs. The magnitude of this response was measured as greater than expected proportions of resting observations on the dark material even at the 25% SAC ratio despite the availability of alternate resting sites, or other behavioral responses such as flight. Similar findings have been described during our experimental hut validation studies in Thailand [Thainchum et al. unpublished data] and Peru [Castro et al. unpublished data] where most *Ae. aegypti* preferred to rest on dark material rather than light, regardless of fabric type even at 25% and 50% SAC.

Although horizontal configuration enhanced resting on both dark cotton and polyester material strips in the current study, as well as under field conditions in experimental huts [Thainchum et al. unpublished data], no consistent preference was observed between upper and lower locations of the dark material within the laboratory assay chamber. This may be due to the relatively small size of the assay that created a spatial bias for the test system – i.e., the laboratory assay dimensions may have precluded substantial differences in height between upper and lower wall portions. However, similar observations have been made in our experimental hut studies where based on observations from upper and lower wall heights, greater proportions of female *Aedes aegypti* populations were observed resting on lower portions of the wall when exposed to cotton material whereas against polyester, upper wall portions were preferred (unpublished data).

An explanation for the variation in resting patterns between the two material types in the current study may include the variation in the microclimate within the test box. Previous studies under laboratory conditions have reported similar findings using *Anopheles* and *Culex* mosquitoes in which they preferred to rest on lower portion of a test box that was cooler than that of the upper portion [47]. Unfortunately, it is not possible to validate this theory using the datasets of the current study because environmental parameters were only measured from a central location inside the bioassay room rather than along the wall surfaces within the test box. Future experiments should integrate microclimate data to better understand behavioral responses.

The fact that cotton enhanced resting on the dark strips as compared to polyester indicates that: 1) the green color of the polyester did not provide as much contrast to the white background as the black color of the cotton; 2) the weave or texture of the cotton provides enhanced tactile cues; or 3) material-specific moisture absorption properties exist under the conditions in which the assay was conducted (i.e. cotton retains more moisture than polyester). When evaluating green cotton versus green polyester simultaneously, cotton still enhanced resting on the dark strips. This finding suggests that the differential resting preference observed between cotton and polyester may not be due to variations in color contrast between material types, but rather is the result of their texture and/or moisture absorption properties. Cotton exhibits greater moisture absorption than that of polyester [48]. It is interesting to note that studies under field conditions in Thailand and Peru are also indicating an overall general decrease in resting when polyester is used versus cotton under chemical-free conditions [Thainchum et al. ; Castro et al. unpublished data]. Such information could be vital in optimizing various vector control tools and could be most beneficial for products designed to target attraction/resting behaviors.

Observations made within the treatment metal boxes during chemical trials indicate that, knockdown responses in all test assays were low (≤5%) even at high chemical dose and treatment area coverage (i.e., 75% and 100%). Low KD even at test doses higher than WHO recommended field application rate for alphacypermethrin (≈7 nm/cm^2^) is probably due to a reduced resting on the treated material and consequentially an increase in proportion of mosquitoes flying (irritated/agitated). It must be noted that test populations were only exposed to the treated surfaces for a total of 10 min, well below the standard 1 hour used in toxicity assays [49]. Also, as the THAI *Ae. aegypti* strain has been characterized as pyrethroid tolerant and DDT resistant [50], [51], it was expected that KD/mortality would be low in these test populations. More importantly, the THAI strain still exhibited a contact irritant response (indicated by increased flying) when exposed to both alphacypermethrin and DDT. These results indicate that sub-lethal approaches to vector control may be effective in resistance management.

Perhaps most important for operational significance is the observation that was made in the test chamber during chemical trials indicating that both mosquito strains continued to rest in greater proportions on dark chemical-treated material versus safe-sites (i.e. chemical-free light material, assay lids and floor) when any dose of either alphacypermethrin or DDT were used. Even under test conditions in which shifting to safe-sites were expected (i.e., 25% SAC), results show no consistent increase in resting counts on chemical-free material. As expected, however, when observations were compared between treatment and matched control assays, significantly fewer mosquitoes were observed resting overall on the dark material treated with chemical.

For all chemical evaluations, the proportion of mosquitoes observed flying was significantly increased in the treatment assay as compared to matched control regardless of the material type used, surface area coverage and configuration of the treated areas within the box. Again, these findings indicate that under current test conditions, *Ae. aegypti* did not simply move to safe-sites (untreated areas) following contact with chemical-treated material but were clearly agitated as measured by an increased flight response. It is this contact irritant response that may elicit escape behavior from a treated space and can be exploited for reducing man-vector contact inside homes. Any residual chemical that is applied to indoor surfaces and has sufficiently strong irritant properties would potentially disrupt the normal resting and may affect the feeding pattern of a vector. These actions could consequently reduce vector – human contact because of rapid escape from inside human dwellings [35]. Such a contact irritant response is well documented in previous field experimentation [32], [52], [53]. While the designs were different in these studies, results from each indicate a rapid escape of mosquitoes from inside experimental huts in response to irritant chemical applications and is the basis for the current laboratory conclusion. The challenge is to ensure that agitation, observed in the current study, does not increase biting on humans prior to escape as this would be counterproductive to intervention impact. Ongoing laboratory studies using the box assay are evaluating escape responses under similar current test conditions to measure the effect of focal treatment on mosquito movement away from a treatment source.

It should be noted that it was not the aim of the current study to compare resting behavior patterns between THAI versus PERU *Ae. aegypti* strains. Each strain was evaluated independently as results from each are currently being validated under field conditions at strain-specific locales (i.e, Kanchanaburi and Iquitos, respectively). However, future studies could investigate the relationship between behavioral phenotype and genetic characteristics of each geographical strain to explore differences that may exist in the resting behavior in response to chemical actions. This information would be useful in understanding the varying challenges in successful implementation of sub-lethal vector control strategies designed to have impact on mosquito populations from different geographic locations.

In summary, results from the current study indicate that both strains of *Ae. aegypti* preferred to rest on dark versus light-colored surfaces during both chemical-free and treated assays, and that agitation (i.e., flight response) was elicited under chemical conditions rather than an increase in resting on untreated safe-sites, even at the lowest 25% D:L coverage. To our knowledge, this is the first attempt to quantify resting responses to sub-lethal doses of irritant chemicals at different treatment surface area coverage. A similar concept of using minimum chemical dose and coverage is also being applied to measure the spatial repellency actions of chemicals to prevent mosquito entry into homes. Pertinent to the larger Push-Pull project under evaluation, laboratory observations have identified those variables that may have the greatest effect in eliciting an escape response following tarsal contact with a chemical-treated surface under experimental conditions. These factors include which material (cotton versus polyester), and configuration (horizontal versus vertical) result in the highest resting response and thereby initiate flight when treated with chemical. Although encouraging, it is the increase in flying that needs to be optimized and to elicit this response in such a way as to minimize opportunities for biting humans. Quantifying vector avoidance of an irritant chemical, through observations of the resting response on untreated and treated surfaces, has been a vital initial component in estimating the likelihood of success of a contact irritant Push-Pull strategy, especially one focused on the use of minimal treatment coverage area. Findings in the current study, together with ongoing field validation, indicate such an approach could be successful.

## Supporting Information

Table S1 Resting observations of *Ae. aegypti* THAI and PERU strains on upper and lower locations of dark material.
(DOC)

Table S2 Resting observations of *Ae. aegypti* THAI strain against green cotton and polyester.
(DOC)

Table S3 Resting observations of *Ae. aegypti* THAI strain against alphacypermethrin treatment conditions.
(DOC)

Table S4 Resting observations of *Ae. aegypti* THAI strain against DDT treatment conditions.
(DOC)

Table S5 Resting observations of *Ae. aegypti* PERU strain against alphacypermethrin and DDT treatment conditions.
(DOC)
