# Supplementary material for: Effects of Irritant Chemicals on Aedes aegypti Resting Behavior: Is There a Simple Shift to Untreated “Safe Sites”?
Source: PLoS Negl Trop Dis. 2011 Jul 26;5(7):e1243. doi: 10.1371/journal.pntd.0001243 (PMC3144182; doi:10.1371/journal.pntd.0001243)
Supplement: Table S1 — Resting observations of Ae. aegypti THAI and PERU strains on upper and lower locations of dark material. (DOC) [file pntd.0001243.s001.doc]

**Table S1.** Resting observations of *Ae. aegypti* THAI and PERU strains on upper and lower locations of dark material.

| Mosquito Strain | Material | Configuration | SAC (%) | Proportion observed resting (%) (N=60) | | P* |
| --- | --- | --- | --- | --- | --- | --- |
|  |  |  |  | Upper | Lower |  |
| THAI | Cotton | N/A | 100 Dark | 55.9 | 44.1 | S |
|  |  |  | 100 Light | 74.2 | 25.8 | S |
|  |  | H | 75 | 33.6 | 66.4 | S |
|  |  |  | 50 | 38.5 | 61.5 | S |
|  |  |  | 25 | 26.9 | 73.1 | S |
|  | Polyester | N/A | 100 Dark | 78.1 | 21.9 | S |
|  |  |  | 100 Light | 73.2 | 26.8 | S |
|  |  | H | 75 | 59.4 | 40.6 | S |
|  |  |  | 50 | 82.4 | 18.1 | S |
|  |  |  | 25 | 72.7 | 27.3 | S |
| PERU | Cotton | N/A | 100 Dark | 50.7 | 49.3 | NS |
|  |  |  | 100 Light | 65.8 | 34.2 | S |
|  |  | H | 75 | 42.0 | 58.0 | S |
|  |  |  | 50 | 28.8 | 71.3 | S |
|  |  |  | 25 | 35.1 | 64.9 | S |
|  | Polyester | N/A | 100 dark | 52.1 | 47.9 | NS |
|  |  |  | 100 Light | 58.4 | 41.6 | S |
|  |  | H | 75 | 61.7 | 38.3 | S |
|  |  |  | 50 | 74.5 | 25.5 | S |
|  |  |  | 25 | 86.7 | 13.3 | S |

* χ2 test P comparing resting on the dark material on upper versus lower regions of the wall

S = P<0.05; NS = P>0.05; N/A = Not applicable; SAC = surface area coverage; H = horizontal; N = 60 from a total of 6 replicates performed for each assay type
