# Supplementary material for: Effects of Irritant Chemicals on Aedes aegypti Resting Behavior: Is There a Simple Shift to Untreated “Safe Sites”?
Source: PLoS Negl Trop Dis. 2011 Jul 26;5(7):e1243. doi: 10.1371/journal.pntd.0001243 (PMC3144182; doi:10.1371/journal.pntd.0001243)
Supplement: Table S2 — Resting observations of Ae. aegypti THAI strain against green cotton and polyester. (DOC) [file pntd.0001243.s002.doc]

**Table S2.** Resting observations of *Ae. aegypti* THAI strain against green cotton and polyester.

| Configuration | SAC (%) | Proportion observed resting (%) | | | | P* |
| --- | --- | --- | --- | --- | --- | --- |
|  |  | Cotton | | Polyester | |  |
|  |  | Dark | Light | Dark | Light |  |
| H | 75 | 84.7 | 11.5 | 75.7 | 16.4 | S |
|  | 50 | 77.2 | 17.3 | 65.8 | 18.5 | NS |
|  | 25 | 77.2 | 16.4 | 62.4 | 27.9 | S |
| V | 75 | 63.2 | 31.2 | 55.3 | 27.8 | NS |
|  | 50 | 69.6 | 23.2 | 60.9 | 23.9 | NS |
|  | 25 | 44.3 | 51.9 | 27.4 | 64.0 | S |

* χ^2^ test P for comparison of resting observation on dark (green) cotton versus dark (green) polyester at each dark:light SAC ratio and each configuration

S = P<0.05; NS = P>0.05; SAC = surface area coverage; H = horizontal; V = vertical; N = 60 from a total of 6 replicates performed for each assay type
