# Supplementary material for: Effects of Irritant Chemicals on Aedes aegypti Resting Behavior: Is There a Simple Shift to Untreated “Safe Sites”?
Source: PLoS Negl Trop Dis. 2011 Jul 26;5(7):e1243. doi: 10.1371/journal.pntd.0001243 (PMC3144182; doi:10.1371/journal.pntd.0001243)
Supplement: Table S3 — Resting observations of Ae. aegypti THAI strain against alphacypermethrin treatment conditions. (DOC) [file pntd.0001243.s003.doc]

**Table S3**. Resting observations of *Ae. aegypti* THAI strain against alphacypermethrin treatment conditions.

| alpha-cypermethrin  doses (nmol/cm^2^) | Material | Configuration | SAC (%) | Proportion observed resting (%) | | P* |
| --- | --- | --- | --- | --- | --- | --- |
|  |  |  |  | Dark | Light |  |
| 2.5 | Polyester | N/A | 100 | 63.3 | N/A | N/A |
|  |  |  |  | N/A | 47.6 | N/A |
|  |  | H | 75 | 50.8 | 0.07 | S |
|  |  |  | 50 | 34.1 | 26.5 | NS |
|  |  |  | 25 | 34.6 | 39.7 | S |
|  |  | V | 75 | 38.0 | 17.3 | S |
|  |  |  | 50 | 26.8 | 25.2 | NS |
|  |  |  | 25 | 28.7 | 39.4 | S |
| 25 | Cotton | N/A | 100 | 79.7 | N/A | N/A |
|  |  |  |  | N/A | 26.1 | N/A |
|  |  | H | 75 | 70.7 | 3.4 | S |
|  |  |  | 50 | 55.2 | 3.8 | S |
|  |  |  | 25 | 50.8 | 26.1 | S |
|  |  | V | 75 | 60.9 | 4.9 | S |
|  |  |  | 50 | 44.8 | 19.1 | S |
|  |  |  | 25 | 44.7 | 35.3 | S |
|  | Polyester | N/A | 100 | 49.3 | N/A | N/A |
|  |  |  |  | N/A | 35.4 | N/A |
|  |  | H | 75 | 37.3 | 9.2 | NS |
|  |  |  | 50 | 55.2 | 3.8 | S |
|  |  |  | 25 | 24.5 | 35.3 | S |
|  |  | V | 75 | 36.7 | 36.0 | S |
|  |  |  | 50 | 24.5 | 42.0 | S |
|  |  |  | 25 | 9.5 | 45.0 | S |

* χ^2^ test P for comparison of resting observation on dark versus light material at each dark:light SAC ratio and each configuration under treatment conditions

S = P<0.05; NS = P>0.05; N/A = Not applicable; SAC = surface area coverage; H = horizontal; V = vertical; N = 60 from a total of 6 replicates performed for each assay type
