# Supplementary material for: Effects of Irritant Chemicals on Aedes aegypti Resting Behavior: Is There a Simple Shift to Untreated “Safe Sites”?
Source: PLoS Negl Trop Dis. 2011 Jul 26;5(7):e1243. doi: 10.1371/journal.pntd.0001243 (PMC3144182; doi:10.1371/journal.pntd.0001243)
Supplement: Table S4 — Resting observations of Ae. aegypti THAI strain against DDT treatment conditions. (DOC) [file pntd.0001243.s004.doc]

**Table S4.** Resting observations of *Ae. aegypti* THAI strain against DDT treatment conditions.

| DDT doses (nmol/cm^2^) | Material | Configuration | | SAC (%) | Proportion observed resting (%) | | P* |
| --- | --- | --- | --- | --- | --- | --- | --- |
|  |  |  | |  | Dark | Light |  |
| 25 | Cotton | N/A | 100 | | 93.5 | N/A | N/A |
|  |  |  |  | | N/A | 45.5 | N/A |
|  |  | H | 75 | | 72.8 | 9.5 | S |
|  |  |  | 50 | | 67.6 | 15.1 | S |
|  |  |  | 25 | | 60.9 | 23.1 | S |
|  |  | V | 75 | | 72.0 | 7.7 | S |
|  |  |  | 50 | | 71.5 | 10.0 | S |
|  |  |  | 25 | | 58.2 | 21.2 | S |
|  | Polyester | N/A | 100 | | 60.1 | N/A | N/A |
|  |  |  |  | | N/A | 62.4 | N/A |
|  |  | H | 75 | | 70.8 | 9.2 | S |
|  |  |  | 50 | | 54.2 | 28.3 | S |
|  |  |  | 25 | | 30.0 | 28.6 | S |
|  |  | V | 75 | | 36.6 | 17.6 | S |
|  |  |  | 50 | | 17.5 | 32.3 | S |
|  |  |  | 25 | | 26.0 | 40.0 | S |
| 250 | Cotton | N/A | 100 | | 85.5 | N/A | N/A |
|  |  |  |  | | N/A | 67.9 | N/A |
|  |  | H | 75 | | 67.8 | 10.2 | S |
|  |  |  | 50 | | 60.8 | 5.4 | S |
|  |  |  | 25 | | 64.4 | 12.5 | S |
|  |  | V | 75 | | 68.1 | 15.9 | S |
|  |  |  | 50 | | 58.1 | 19.4 | S |
|  |  |  | 25 | | 42.8 | 39.6 | S |
|  | Polyester | N/A | 100 | | 58.4 | N/A | N/A |
|  |  |  |  | | N/A | 63.7 | N/A |
|  |  | H | 75 | | 42.1 | 4.9 | S |
|  |  |  | 50 | | 58.3 | 14.2 | S |
|  |  |  | 25 | | 18.5 | 21.2 | S |
|  |  | V | 75 | | 29.1 | 24.6 | S |
|  |  |  | 50 | | 26.1 | 29.6 | NS |
|  |  |  | 25 | | 20.7 | 43.4 | S |

* χ^2^ test P for comparison of resting observation on dark versus light material at each dark:light SAC ratio and each configuration under treatment conditions

S = P<0.05; NS = P>0.05; N/A = Not applicable; SAC = surface area coverage; H = horizontal; V = vertical; N = 60 from a total of 6 replicates performed for each assay type
