# Supplementary material for: Effects of Irritant Chemicals on Aedes aegypti Resting Behavior: Is There a Simple Shift to Untreated “Safe Sites”?
Source: PLoS Negl Trop Dis. 2011 Jul 26;5(7):e1243. doi: 10.1371/journal.pntd.0001243 (PMC3144182; doi:10.1371/journal.pntd.0001243)
Supplement: Table S5 — Resting observations of Ae. aegypti PERU strain against alphacypermethrin and DDT treatment conditions. (DOC) [file pntd.0001243.s005.doc]

**Table S5.** Resting observations of *Ae. aegypti* PERU strain against alphacypermethrin and DDT treatment conditions.

| Chemicals | Dose (nmol/cm^2^) | Material | Configuration | SAC (%) | Proportion observed resting (%) | | P* |
| --- | --- | --- | --- | --- | --- | --- | --- |
|  |  |  |  |  | Dark | Light |  |
| alphacypermethrin | 25 | Cotton | N/A | 100 | 63.4 | N/A | N/A |
|  |  |  |  |  | N/A | 25.1 | N/A |
|  |  |  | H | 75 | 77.1 | 4.2 | S |
|  |  |  |  | 50 | 64.4 | 11.9 | S |
|  |  |  |  | 25 | 48.9 | 25.7 | S |
|  |  |  | V | 75 | 71.0 | 5.2 | S |
|  |  |  |  | 50 | 59.4 | 21.8 | S |
|  |  |  |  | 25 | 36.6 | 21.1 | S |
|  |  | Polyester | N/A | 100 | 35.4 | N/A | N/A |
|  |  |  |  |  | N/A | 32.5 | N/A |
|  |  |  | H | 75 | 39.7 | 9.3 | NS |
|  |  |  |  | 50 | 21.4 | 20.1 | NS |
|  |  |  |  | 25 | 24.4 | 23.1 | S |
|  |  |  | V | 75 | 26.4 | 27.1 | S |
|  |  |  |  | 50 | 20.9 | 23.6 | NS |
|  |  |  |  | 25 | 18.7 | 39.7 | S |
| DDT | 25 | Cotton | N/A | 100 | 79.0 | N/A | N/A |
|  |  |  |  |  | N/A | 44.5 | N/A |
|  |  |  | H | 75 | 71.2 | 4.7 | S |
|  |  |  |  | 50 | 62.5 | 24.3 | S |
|  |  |  |  | 25 | 56.9 | 33.8 | S |
|  |  |  | V | 75 | 72.8 | 9.5 | S |
|  |  |  |  | 50 | 57.9 | 25.2 | S |
|  |  |  |  | 25 | 58.8 | 29.9 | S |
|  |  | Polyester | N/A | 100 | 62.7 | N/A | N/A |
|  |  |  |  |  | N/A | 65.9 | N/A |
|  |  |  | H | 75 | 64.1 | 17.3 | NS |
|  |  |  |  | 50 | 69.0 | 8.3 | S |
|  |  |  |  | 25 | 30.0 | 28.6 | S |
|  |  |  | V | 75 | 47.4 | 17.4 | S |
|  |  |  |  | 50 | 35.9 | 37.6 | S |
|  |  |  |  | 25 | 26.0 | 40.0 | S |
|  | 250 | Cotton | N/A | 100 | 65.7 | N/A | N/A |
|  |  |  |  |  | N/A | 51.7 | N/A |
|  |  |  | H | 75 | 73.3 | 5.5 | S |
|  |  |  |  | 50 | 66.6 | 13.6 | S |
|  |  |  |  | 25 | 64.3 | 11.7 | S |
|  |  |  | V | 75 | 55.6 | 20.0 | S |
|  |  |  |  | 50 | 68.9 | 20.7 | S |
|  |  |  |  | 25 | 47.1 | 33.3 | S |
|  |  | Polyester | N/A | 100 | 58.3 | N/A | N/A |
|  |  |  |  |  | N/A | 48.5 | N/A |
|  |  |  | H | 75 | 48.3 | 12.7 | S |
|  |  |  |  | 50 | 46.3 | 23.3 | S |
|  |  |  |  | 25 | 26.1 | 54.2 | S |
|  |  |  | V | 75 | 50.0 | 13.1 | S |
|  |  |  |  | 50 | 47.5 | 28.8 | S |
|  |  |  |  | 25 | 31.3 | 52.7 | S |

* χ^2^ test P for comparison of resting observation on dark versus light material at each dark:light SAC ratio and each configuration under treatment conditions.

S = P<0.05; NS = P>0.05; N/A = Not applicable; SAC = surface area coverage; H = horizontal; V = vertical; N = 60 from a total of 6 replicates performed for each assay type
